# Comparative effectiveness of prophylactic hyperthermic intraperitoneal chemotherapy (HIPEC) for resected low-grade appendiceal mucinous neoplasm (LAMN): A protocol for systematic review and network meta-analysis

**DOI:** 10.1097/MD.0000000000022071

**Published:** 2020-09-04

**Authors:** Wenming Yang, Pan Nie, Xueting Liu, Jikui Peng

**Affiliations:** a Department of Gastrointestinal Surgery, West China Hospital of Sichuan University, Chengdu; b Department of Gastrointestinal Surgery, Inner Mongolia People's Hospital, Hohhot; c Department of Gastrointestinal Surgery, The Third Affiliated Hospital of Sun Yat-Sen University, Guangzhou; d Department of Evidence-Based Medicine and Clinical Epidemiology, West China Hospital of Sichuan University, Chengdu, China.

**Keywords:** appendiceal mucinous neoplasm, effectiveness, hyperthermic intraperitoneal chemotherapy, network meta-analysis, RCT

## Abstract

**Background:**

Whether prophylactic hyperthermic intraperitoneal chemotherapy (HIPEC) offers long-term survival benefit to patients with low-grade appendiceal mucinous neoplasms (LAMNs) after resection surgery is still under heated debate. The aim of the present meta-analysis is to investigate the comparative effectiveness and safety of prophylactic HIPEC regimens in LAMNs

**Methods:**

A systematic search of MEDLINE, EMBASE, PubMed, Web of Science, the Cochrane Central Register of Controlled Trials, International Clinical Trials Registry Platform (ICTRP), clinicaltrials.gov and controlledtrials.com will be performed. All published RCTs and quasi-RCTs through July 20, 2020 with language restricted in English will be included in this review study. Two reviewers will independently conduct the procedures of study identification, data collection, and methodological quality assessment. The primary outcomes are overall survival (OS) and disease-free survival (DFS). The secondary outcomes consist of peritonitis and sepsis, colonic fistula, chemotherapy-associated adverse events, and adhesive intestinal obstruction. The pooled odds ratios (ORs) or hazard ratios (HRs) and relative 95% confident intervals (CIs) of each outcome measurement will be calculated. EndNote X9 software will be applied to manage all citations. The Stata software version 14.0 and R x64 software version 3.5.1 will be employed for main statistical analyses.

**Discussion:**

This study will employ a network meta-analysis to summarize direct and indirect evidence in the specific area to provide detailed individualized guidance on surgical management for LAMNs.

**Registration:**

This protocol was registered with the International Platform of Registered Systematic Review and Meta-Analysis Protocols (INPLASY) on 25 July 2020 (registration number *INPLASY202070112*).

## 1 Introduction

Appendiceal mucinous neoplasms (AMNs) are relatively rare tumors which account for less than 2% of appendectomies.^[1]^ The World Health Organization classifies the majority of noninvasive epithelial lesions originated from appendix as low-grade appendiceal mucinous neoplasms (LAMNs).^[2]^ Histologically, LAMNs are characterized by well-differentiated adenomas which can proliferate outside the appendix in a biological malignant fashion. LAMNs may perforate and spread throughout the peritoneal cavity resulting in the distinctive and frequently aggressive syndrome called pseudomyxoma peritonei (PMP).^[3]^ Due to high relapse risk and poor 10-year overall survival (OS) rate after treatment, PMP should be also regarded as malignancy.^[4,5]^ It is suggested that right hemicolectomy confers minimal survival benefit and is reserved for certain cases such as positive resection margins and lymph nodes after appendectomy or perforated appendix.^[6–8]^ Conversely, there are some medical centers advocating aggressive treatment approaches with prophylactic hyperthermic intraperitoneal chemotherapy (HIPEC) as an effective means to prevent from development into widespread PMP.^[9,10]^ However, there is no existing consensus on optimal HIPEC regimens due to lack of pairwise and network meta-analyses (NMAs) of head-to-head randomized controlled trials (RCTs). As a novel therapy, the efficacy and safety of prophylactic HIPEC regimen requires further and more careful assessment. Thus, the aim of our meta-analysis is to investigate the comparative effectiveness and safety of enrolled prophylactic HIPEC regimens in patients with LAMNs after resection surgery.

## 2 Methods

This protocol was registered at the International Platform of Registered Systematic Review and Meta-Analysis Protocols (INPLASY) on 25 July 2020 with registration number *INPLASY202070112* (https://inplasy.com/inplasy-2020-7-0112/). This protocol is designed under the guidance of Preferred Reporting Items for Systematic Review and Meta-Analysis Protocols (PRISMA-P) checklist.^[11,12]^ The ethical approval is not required due to the nature of meta-analysis.

### 2.1 Eligibility criteria

The detailed eligibility criteria are summarized in accordance with the PICOs search tool (population, intervention, comparison, outcome, and study design).

### 2.2 PICO

P: patients with LAMNs after resection surgery;

I: prophylactic HIPEC;

C: follow-up and surveillance;

O: prognostic effectiveness.

### 2.3 Participants

Patients with histopathologically confirmed LAMNs and no peritoneal involvement after appendectomy or extended resection surgery will be included. The surgical decision might be made according to the diagnosis of acute appendicitis or abnormal appendix seen during colonoscopy or unrelated operation. The operation might be performed regardless of under the condition of open or laparoscopy.

### 2.4 Comparison of interventions

The prophylactic HIPEC after resection surgery would be administrated for patients in the HIPEC group as soon as the histopathologic diagnosis was established. Patients in the Follow-up group would be scheduled to postoperative routine follow-up and surveillance, including serum tumor markers (CEA, CA19-9, CA125), imaging (Computer Tomography of chest, Computer Tomography with contrast agent or Magnetic Resonance Imaging of abdomen and pelvis), and biopsy when necessary.

### 2.5 Outcome measurements

The primary outcomes are overall survival (OS) and disease-free survival (DFS). OS is defined as the time from randomization until death from any cause. DFS is defined as the time from randomization until recurrence, metastasis, or occurrence of pseudomyxoma peritonei (PMP) confirmed by imaging, laparoscopic exploration and biopsy. The secondary outcomes consist of peritonitis and sepsis, colonic fistula, chemotherapy-associated adverse events, and adhesive intestinal obstruction.

### 2.6 Study design

Published eligible RCTs and quasi-RCTs will be enrolled in the meta-analysis. The sequence generation, blinding, and allocation concealment should be explicitly described. Only articles originally written in English or translated into English will be considered.

### 2.7 Information sources and search strategy

A systematic search of MEDLINE, Excerpta Medica Database (EMBASE), PubMed, Web of Science, and the Cochrane Library databases will be performed. The Cochrane Central Register of Controlled Trials, International Clinical Trials Registry Platform (ICTRP), clinicaltrials.gov and controlledtrials.com will be also searched for ongoing trials. The relative references, academic conferences and network resources in the included literature will be further screened for potential eligible ones. When multiple reports describing the same sample were published, the most recent or complete report will be included. All RCTs published in electronic databases through July 20, 2020 with language restricted in English will be included in this review study.

The search strategy on PubMed will be as follows:

#1 (((((((appendiceal mucinous neoplasm) OR appendiceal mucinous tumor) OR appendiceal mucocele) OR appendiceal mucinous cystadenoma) OR appendiceal neoplasia) OR appendiceal cancer) OR appendicular tumor) OR appendix tumors
#2 ((((intraperitoneal perfusion) OR “peritoneal perfusion”) OR hyperthermic intraperitoneal chemotherapy) OR “heated intraperitoneal chemotherapy”) intraperitoneal chemotherapy
#3 ((((((((“Randomized Controlled Trial” [Publication Type]) OR “Controlled Clinical Trial” [Publication Type]) OR “randomized” [tiab]) OR “placebo” [tiab]) OR “Clinical Trials as Topic”[Mesh: NoExp]) OR “randomly” [tiab]) OR “trial” [ti])) NOT ((“Animals” [mh]) NOT “ humans” [mh])
#4 #1 AND #2 AND #3

This search strategy will be modified to be suitable for other certain electronic databases.

### 2.8 Study selection and data collection

#### 2.8.1 Study selection

**Figure 1:**
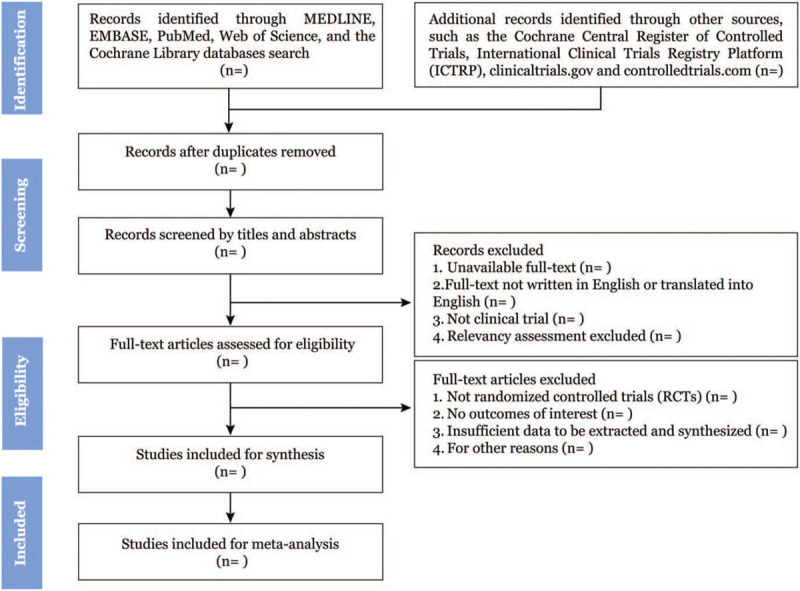
Flow diagram of study selection.

Two authors (WY, PN) will screen all searched titles and abstracts independently for eligibility and relevance to this review study. EndNote X9 software (Clarivate Analytics) will be employed to manage all citations, as well as for duplicates removing. The study selection procedure is summarized in a PRISMA-P flow diagram as shown (Fig. 1). All studies meeting the exclusion criteria, such as case reports, letters, conference summaries, will be removed with recorded reasons firstly. Then full-texts of studies will be investigated carefully based on the inclusion criteria. Finally, review authors will discuss any disagreements and involve the third party (JP) to help resolve remaining conflicts.

#### 2.8.2 Data extraction and collection

Two authors (WY, PN) will independently extract relevant information from each eligible study using a standardized form. Information about general characteristics (source, region, year of publication, title, first author, study type), data for methodological quality assessment, participants’ characteristics in each group (HIPEC vs Follow-up), median follow-up period, and all outcomes of interest will be included. variables. Hazard ratios (HRs) will be extracted the reported values from studies or be estimated from survival curves by established methods. The log hazard ratio (lnHR) and its relevant standard error (SE) will be calculated by approximating the data of the Kaplan-Meier survival curve from original articles utilizing Engauge Digitizer version 4.1 (Free Software Foundation, Inc., Boston, Massachusetts, USA) and processing the data via the Calculations Spreadsheet in Microsoft Excel proposed by Tierney et al.^[13,14]^ In addition, we will contact the corresponding author by e-mail to request for sufficient original data to ensure accuracy in this meta-analysis. Then the cross-checked data will be entered into the Stata software version 14.0 (Stata Corp LP, College Station, TX) for high-quality management and subsequent data synthesis.

#### 2.8.3 Risk of bias

The methodological quality will be assessed by two review authors (WY, PN) utilizing the RevMan software version 5.3 (The Nordic Cochrane Centre, The Cochrane Collaboration, Copenhagen, Denmark) ‘Risk of Bias’ (RoB) assessment tool in terms of selection bias (method of randomization and allocation concealment), information bias (masking of outcome adjudicators), and bias in the analysis (intention to treat analysis and completeness of follow-up). Risk of bias for each study will be quantified in adherence to the criteria outlined in the Cochrane Handbook for Systematic Reviews of Interventions.^[15]^ Disagreement between two reviewers will be resolved by discussion and consulting an expert (XL) in Evidence-Based Medicine (EBM). The RoB table and graph will be drawn by RevMan 5.3.

### 2.9 Data synthesis and statistical analysis

#### 2.9.1 Pairwise meta-analysis

The Stata software version 14.0 will be used to perform pairwise meta-analyses. Pooled HRs and relative 95% confidence intervals (CIs) will be calculated for time-to-event outcomes (OS, DFS); pooled odds ratios (ORs) with 95% CIs will be calculated for dichotomous outcomes (peritonitis and sepsis, colonic fistula, chemotherapy-associated adverse events, adhesive intestinal obstruction). Statistical significance will be set at *P* < .05 to summarize the findings across the studies. Considering heterogeneity between studies, pooled analyses will be conducted with a random effect model (REM) rather than a fixed effect model (FEM). Statistical heterogeneity between studies will be evaluated using the chi-square (χ^2^) test and quantified with Cochrane's Inconsistency (*I*^2^)-statistic. We set 50% as a cut-off value, such that *P* value <.10 and/or *I*^2^ > 50% are considered substantial heterogeneity. Sensitivity analysis and subgroup analysis will be set up to explore the sources of heterogeneity among the included studies. The possibility of publication bias will be assessed primarily by visual analysis of Begg funnel plot. The Egger test will be applied to identify further potential publication bias when necessary.

#### 2.9.2 Network meta-analysis

A Bayesian network NMA will be conducted using the R x64 software version 3.5.1. The inconsistency between direct and indirect comparisons will be tested using node splitting method if a loop exists.^[16]^ Surface under the cumulative ranking area (SUCRA) will be used to rank the different HIPEC regimens for patients with resected LAMNs. Comparison-adjusted funnel plots will be generated to detect the small sample effects on the results. A network plot will be conducted to present the comparisons of the treatments among trials to ensure whether an NMA is feasible. As for network geometry, nodes represent different treatments and size of node represents sample sizes of intervention; edges represent the head-to-head treatments and thickness of edge represents numbers of included studies. All the result figures will be drawn using R x64 and Stata software.

#### 2.9.3 Subgroup analysis and sensitivity analysis

Subgroup analyses will be conducted to identify possible sources of heterogeneity on the basis of sex, age, region, history of colorectal cancer, selective or emergency surgery, and appendectomy or extended resection surgery. The sensitivity analysis will be performed to ensure the stability of measure effects of primary outcomes by removing one by one those studies with suspected high risk of bias in terms of sample size, study design, heterogeneity qualities, and with non-informative prior distributions for the heterogeneity parameters. Non-robust results of primary outcomes identified by sensitivity analysis will be added to a descriptive analysis.

#### 2.9.4 Quality of evidence

The quality of evidence regarding all outcomes will be assessed using the Grading of Recommendations Assessment, Development, and Evaluation (GRADE) which mainly contains several dimensions, such as risk of bias, inaccuracy, inconsistency, indirectness, and publication bias.^[17]^ The strength of the body of evidence will be graded into 4 levels: very low, low, moderate, and high level.

## 3 Discussion

Currently, whether prophylactic HIPEC offers long-term survival benefit to patients with resected LAMNs is still under heated debate. This study will employ an NMA to summarize direct and indirect evidence in the specific area. The results are expected to provide evidence-based individualized guidance on surgical management for LAMNs.

## Author contributions

**Conceptualization:** Wenming Yang, Jikui Peng.

**Software and data curation:** Pan Nie, Xueting Liu.

**Methodology and statistical analysis:** Wenming Yang, Jikui Peng, Xueting Liu.

**Supervision and validation:** Jikui Peng.

**Writing – original draft:** Wenming Yang, Pan Nie.

**Writing – review & editing:** Jikui Peng.
